# Acute Myeloid Leukaemia: New Targets and Therapies

**DOI:** 10.3390/ijms18122577

**Published:** 2017-11-30

**Authors:** Geoffrey Brown, Ewa Marcinkowska

**Affiliations:** 1 Institute of Clinical Science, College of Medical and Dental Sciences, University of Birmingham, Edgbaston, Birmingham B15 2TT, UK; 2 Institute of Immunology and Immunotherapy, College of Medical and Dental Sciences, University of Birmingham, Edgbaston, Birmingham B15 2TT, UK; 3 Laboratory of Protein Biochemistry, Faculty of Biotechnology, University of Wroclaw, Joliot-Curie 14a, 50-383 Wroclaw, Poland; ema@cs.uni.wroc.pl

The most common acute hematological malignancy in adults is acute myeloid leukaemia (AML), accounting for more than 80% of cases in patients over 60 years of age [1]. AML is the second most common acute leukaemia in children, accounting for 15–20% of leukaemia cases [2,3]. Morphological classification of AML into eight sub-types (FAB M0–M7) based on the type of cell from which the leukemia developed, and on its degree of maturity, was in use until the end of the last century [4]. A new World Health Organization (WHO) classification introduced four main AML groups, based on cytogenetic abnormalities, and is important to the approach used to treat this disease [5]. In particular, acute promyelocytic leukaemia (APL), a distinct M3 subtype of AML, was once an incurable disease and the use of all-*trans* retinoic acid (ATRA)-based differentiation therapy and anthracycline-based chemotherapy now provides a cure rate above 80% (reviewed in [6]). However, aside from APL, around 90% of older patients (aged ≥ 60 years) and more than half of young adult patients (aged < 60 years) die from their disease [7], and AML accounts for the largest number of annual deaths due to leukaemias in the US [2]. The survival of older patients who are unable to tolerate chemotherapy is dismal, at only five to 10 months [8], and complete remission rates for relapsed patients are <25% [9,10]. 

The Fms-like tyrosine kinase 3 (FLT3), a class III receptor tyrosine kinase, and FLT3 ligand (FL) play an important role in the proliferation, differentiation, and survival of hematopoietic cells [11]. Mutated FLT3 is expressed in a subset of AML patients and confers a poor prognosis. The most common mutation, which occurs in about 25% of AML patients, is an internal tandem duplication (ITD) in the juxtamembrane region of FLT3, which causes ligand-independent dimerization and constitutive receptor activation [12]. Point mutations in the tyrosine kinase domain (TKD), FLT3-TKD, occur in approximately 7% of AMLs [13]. FLT3 tyrosine kinase inhibitors are well tolerated, as a monotherapy and with intensive chemotherapy, and second generation inhibitors have shown significant promise as a treatment for relapsed and refractory AML. The paper by Mooney and colleagues [14] and the review by Tsapogas and colleagues [15] advance the knowledge of FLT3 and FL. Mooney and colleagues have examined the expression of mRNA FLT3 (mRNA and cell surface protein) by hematopoietic stem cells (HSC) and various progenitor cells (HPC). A sub-population of short-term and long-term HSC express FLT3. As expected, expression by HPC was observed for these cells with lympoid, granulocytic, and myelomomocytic potential. Regarding FLT3 expression by HSC, Tsapogas provides evidence to support an instructive role of FL signalling at early stages of hematopoiesis, in addition to a role in promoting cell survival and proliferation. The precise pattern of expression of FLT3 by HSC/HPC and the roles of FL in normal hematopoiesis are critical to a better understanding of AML subtypes, the process of disease progression, as well as for the development of therapeutic strategies to target FLT3-mutated AML.

Regarding other kinase inhibitors, T315, an inhibitor of integrin-linked kinase, has been shown to suppress the proliferation of breast and stomach cancer cells and chronic lymphocytic leukaemia cells. Chiu and collegues [16] report that this agent is cytotoxic against the AML cell lines HL-60 and THP-1 and primary leukaemia cells from AML patients. T315 also suppresses the growth of THP-1 xenografts. Chiu and colleagues decribe aspects of the mechanism of action of T315, in driving apoptosis and autophagic cell death, and suggest further assessment of the use in treating AML and other leukaemias. However, there is the need to modify T315 to increase efficacy and reduce toxicity. Apoptosis can also be induced in human leukaemia cells by the curcumin analogue EF-24. In their study, Skoupa and colleagues [17] investigate the mechanism by which EF-24 induces cell death in K562 cells. They propose that EF-24 may be suitable as an anticancer agent, specifically in cases of drug resistance.

The oncogenic or chromosomal mutations that are present in a patient’s AML cells at diagnosis are important to personalized treatment. The best known is a balanced translocation between chromosomes 15 and 17 [t(15;17)(q22;q21)] resulting in the formation of promyelocytic leukemia (PML) and retinoic acid receptor alpha (RARα) fusion protein [18]. Leukaemic blasts which carry this mutation are susceptible to ATRA-induced cell differentiation. Watts and collegues [19] report a salutary lesson from the treatment outcome of a case of AML. The case is characterised by a new t(4;15)(q31;q22) translocation, resulting in the expression of the TMEM154-RASGRF1 fusion protein. The patient who was treated with ATRA as a part of a clinical study died from rapid disease progression. An increase in the expression of RARγ was observed upon treatment of the patient’s cells ex vivo with ATRA, and they proliferated in response to ATRA and a RARγ agonist. The disease progression could be related to an increase in RARγ, which plays a role in hematopoietic stem cell self-renewal and proliferation. Furthermore, there are implications for the use of retinoid-based differentiation therapy for certain cases. 

In addition to ATRA, the differentiating agent 1,25-dihydroxyvitamin D (1,25D) has been reported to have beneficial effects in combination therapy for cancer. The paper by Janik and colleagues [20] examines the regulation of the vitamin D receptor (*VDR*) gene by 1,25D and ATRA in blood cells at early stages of their differentiation. ATRA, but not 1,25D, upregulates the expression of *VDR* in human early-stage blood cells. As to early-stage mouse cells, *VDR* is upregulated by 1,25D, but not by ATRA. Hence, *VDR* regulation in humans and mice is different, which is germane to testing combinations of agents for use in differentiation therapy. The findings also bring to attention that the level of expression of VDR protein is low in patients’ AML blasts that do not respond to 1,25D. The level can be upregulated by ATRA treatment and this is relevant to the combined therapeutic use of ATRA and 1,25D. 

There is an urgent need for new treatments for AML, which will include the need to identify new molecules to target. A number of recurrent mutations in AML involve genes concerned with regulating the epigenetic landscape [21]. Gain or loss of function of the gene encoding the EZH2 methytransferase occurs in various malignancies. Sbirkov and colleagues [22] describe the use of affinity-purification mass spectrometry to identify new partners of EZH2 and potential new non-histone substrates. Of particular note is that EZHZ has a role in regulating translation, via interacting with RNA binding proteins and methylating eEF1A1. eEF1A1 is a component of protein synthesis, highly expressed in tumours, and therapeutic targeting is a possibility in AML. The review by Gbolahan and colleagues [23] focusses on the benefit of immunotherapeutic interventions to the long-term control of AML, including the use of hypomethylating agents. The results from early phase immunotherapeutic interventions, for example, the use of a monoclonal antibody to CD33 which is highly expressed on AML blasts, are encouraging. The interleukin-3 receptor α chain (CD123), FcγRI (CD64) and C-type lectin-like molecule 1 are also promising targets. The review considers the use of hypomethylating agents to increase the antigenicity of AML cells and their role as immunomodulatory drugs.

The articles by Player et al., Khan et al., and Almeida et al. describe the outcomes from clinical studies. Player and colleagues [24] examine azacitidine for front-line therapy of patients with AML, by reference to international phase 3 trial data and data from the Austrian Azacytidine registry. The authors report clinically-meaningful improvement in overall survival for patients treated with front-line azacytidine versus conventional regimens, and conlude that azacytidine appears efficacious as a front-line treatment for WHO-AML patients. Khan and colleagues [25] examine the clinical outcomes in patients with *RUNX1*-mutated AML. The response of older patients to treatment with hypomethylating agents and survival were found to be independent of *RUNX1* status, leading the authors to conclude that future studies should focus on the prognostic value of *RUNX1* mutations relative to co-occuring mutations. Almeida and colleages [26] examine the outcomes from the various types of treatment for acute erythroleukemia (AEL). AEL typically has a poor prognosis. From a comparison of patients treated with hypomethylating agents, front-line, and intensive chemotherapy, the results provide support to the use of hypomethylating agents to treat AEL. However, high-risk patients treated first-line with hypomethylating agents live just a few months longer (13.3 versus 7.5 for patients treated with intensive chemotherapy).

In summary, there is quite some way to go to extend the success in treating APL to other types of AML. As mentioned above, 90% of older patients die from their disease. In populations of developed countries, cancers are turning into prevalent diseases of the aged. According to epidemiological data, about 80% of cancers are diagnosed in patients older than 55, with the median age at diagnosis above 60 years [27]. This is also the case for AML for which the median age at diagnosis is 67 years [28]. Many older AML patients are not able to receive intensive chemotherapy. Hence, there is the need for new approaches to the treatment of AML, and other malignancies, in elderly patients. New therapeutic targets are important, as is consideration of differentiation therapy, including agents that modify the epigenetic status of cells, combined with gentler chemotherapy. A means of preventing AML relapse and holding in check for life will provide a better quality of life to elderly patients.
